# Antimicrobial Resistant *Salmonella* in Canal Water in Bangkok, Thailand: Survey Results Between 2016 and 2019

**DOI:** 10.3390/ijerph22091333

**Published:** 2025-08-27

**Authors:** Saowapa Khotchalai, Fuangfa Utrarachkij, Angkana Lekagul, Wanwisa Kaewkhankhaeng, Viroj Tangcharoensathien

**Affiliations:** 1 Department of Microbiology, Faculty of Public Health, Mahidol University, Bangkok 10400, Thailand; fuangfa.utr@mahidol.ac.th; 2 International Health Policy Program Foundation, Nonthaburi 11000, Thailand; angkana@ihpp.thaigov.net (A.L.); wanwisa@ihpp.thaigov.net (W.K.); viroj@ihpp.thaigov.net (V.T.)

**Keywords:** antimicrobial resistance, multidrug-resistant, *Salmonella* spp., One Health, environment health, urban canals, wastewater, ESBL, integron

## Abstract

Antimicrobial resistance (AMR) in environmental reservoirs is an emerging global health concern, particularly in urban settings with inadequate wastewater management. This study aimed to investigate the prevalence and resistance profiles of *Salmonella* spp. in canal water in Bangkok and assess the distribution of key antibiotic resistance genes (ARGs). Between 2016 and 2019, a total of 1381 water samples were collected from 29 canals. *Salmonella* spp. were isolated using standard microbiological methods and tested for susceptibility to 13 antibiotics. Polymerase chain reaction (PCR) was used to detect extended-spectrum β-lactamase (ESBL) genes and class 1 integron. *Salmonella* was found in 89.7% of samples. Among these, 62.1% showed resistance to at least one antimicrobial, and 54.8% were multidrug-resistant (MDR). The highest resistance was observed against streptomycin (41.4%). ESBL genes, predominantly *blaCTX-M*, were detected in 72.2% of tested isolates, while class 1 integrons were found in 67.8%, indicating a strong potential for gene dissemination. The results highlight urban canals as critical environment reservoirs of AMR *Salmonella* serovars, posing significant public health risks, particularly where canal water is used for agriculture, household, or recreational purposes. Strengthened environmental surveillance and effective wastewater regulation are urgently needed to mitigate AMR bacteria transmission at the human–environment–animal interface.

## 1. Introduction

Antimicrobial resistance (AMR) is an increasing major public health concern worldwide caused by excessive use of antibiotics in humans and agriculture for therapeutic and nontherapeutic purposes [1]. It claims more than 10 million deaths annually worldwide and may push 24 million people into severe poverty by 2050 [2].

A major concern is the release of AMR bacteria into the environment and food chain [3]. The One Health approach, recognizing the inter-connectedness of humans, animals, and the environment, is a vital mechanism in addressing AMR [4,5,6]. The Thai National Strategic Plan on Antimicrobial Resistance (2017–2021) was adopted and implemented with the aim of achieving a 20% reduction in antimicrobial consumption in the human sector and a 30% reduction in the animal sector through the One Health approach [7]. Under Strategy I, the plan seeks to develop a national integrated AMR surveillance system covering humans, animals, and the environment; however, progress in the environmental sector has been comparatively slow.

In the environment, soil and water serve as reservoirs for antibiotic residues and AMR bacteria [8]. One of the primary pathways for AMR bacteria contamination in the environment is through untreated waste and wastewater from households, hospitals, animal farms, and natural waterways [9]. Successful mitigation strategies to reduce AMR bacteria in environmental areas include (i) avoiding the creation of settings that select for, mobilize, and allow persistence of resistance genes in bacterial communities, (ii) reducing transmission routes for resistant bacteria from the diseased human’s (or animal’s) microbiome to environment, and (iii) limiting the selection pressure for resistant pathogens through prudent use of antibiotics for humans and animals.

*Salmonella enterica* subsp. *enterica* is responsible for the majority of *Salmonella* infections in humans and other warm-blooded animals, exhibits a ubiquitous distribution, and is frequently isolated from aquatic environments [10,11]. This pathogen is typically present in untreated sewage with large numbers of 10^3^–10^4^ colony-forming units per liter (CFU/L) and can be detected in effluent drains even after secondary advanced treatments [12,13]. Although most *Salmonella* infections are foodborne, cases of salmonellosis caused by contaminated recreational water and surface water have been reported [14]. While most *Salmonella* infections are self-limiting, antimicrobial treatment is necessary for invasive infections or severe diarrhea. The World Health Organization (WHO) recommends fluoroquinolones (FQs) and third-generation cephalosporins as choices for the treatment of salmonellosis, which are classified as critically important for human medicine [15]. However, these are extensively used in veterinary practices [16,17,18]. The use of antimicrobial agents in livestock production and in humans has resulted in the survival of AMR *Salmonella* isolates from different sources, which have been frequently reported worldwide [19].

Bangkok, the capital city of Thailand, has an extensive canal network surrounded by residential, farming, and small agricultural areas. Household and agricultural waste potentially releases AMR bacteria and antibiotic residues into the canal system. Despite growing concerns regarding AMR contamination in urban waterways, data on the presence and spread of AMR *Salmonella* in Bangkok’s canal network remains limited. Given that these canals are directly connected to communities and often serve for irrigation, aquaculture, and even informal recreational use, pathogen surveillance in canal water is essential for early detection of AMR threats and for guiding targeted public health interventions.

This study aims to investigate the occurrence of *Salmonella* spp., their AMR profiles, and antibiotic resistance genes (ARGs) in canal water in Bangkok. The findings are expected to provide a clearer understanding of the current situation and support data for the development of targeted mitigation policies and interventions to address AMR in the environment.

## 2. Materials and Methods

### 2.1. Sample Collection

**Figure 1:**
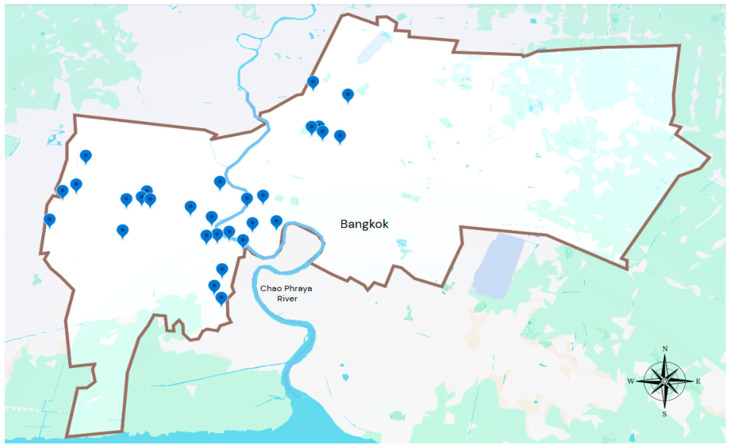
Sampling locations of canal water in Bangkok, Thailand (2016–2019). Blue markers represent the 29 canal sampling sites.

A total of 1392 canal water samples were collected from 29 canals in Bangkok, Thailand, between 2016 and 2019 (Figure 1). Sampling was conducted by the Metropolitan Water Quality Control Division, the agency responsible for routine wastewater quality monitoring in Bangkok. The Bangkok Metropolitan Area has a population of approximately 10.5 million inhabitants, and more than 150 public and private hospitals are served by multiple wastewater treatment plants (WWTPs) of varying capacities. The selection of these 29 canals was based on their geographic distribution across different districts, their proximity to residential, agricultural, and industrial areas, and historical records of water quality concerns from the metropolitan monitoring program. Monthly sampling was systematically performed at all sites throughout the four-year study period (2016–2019), providing consistent temporal coverage across wet and dry seasons, a broad spatial representation of urban, agricultural, and industrial settings, and a sufficiently large sample size. This longitudinal and geographically stratified design yielded a robust dataset for reliable trend analysis, resistance profiling, and molecular characterization, ensuring strong validity for environmental surveillance.

Bangkok’s canal network is closely linked to surrounding communities and is used for irrigation, aquaculture, and informal recreation, creating direct interfaces for human, animal, and environmental health risks. In densely populated urban areas, such canals can serve as critical reservoirs and transmission pathways for antimicrobial-resistant pathogens. Surveillance of pathogens in canal water is therefore essential for early detection of AMR threats and for informing targeted public health interventions, especially in rapidly urbanizing cities like Bangkok, where environmental AMR monitoring remains underdeveloped.

For each 200 mL water sample collection, the bottle was submerged facing upstream to a depth of approximately 20 cm below the water surface before the cap was removed underwater to allow natural filling into the bottle [20]. The sample was immediately placed in an ice box to maintain a cool temperature and transported to the Department of Microbiology, Faculty of Public Health, Mahidol University, for processing on the same day. Eleven of the samples were excluded from the study due to inadequate water quality for analysis after laboratory arrival.

### 2.2. Isolation and Identification of Salmonella

The bottles containing water samples were vigorously shaken, and then 50 mL of each water sample was mixed with an equal volume of double-strength buffered peptone water and incubated at 37 °C for 18 h. The enriched sample was streaked onto Modified Semisolid Rappaport–Vassiliadis (MSRV) medium and incubated at 42 °C for 18 h. Presumptive *Salmonella* colonies growing on the MSRV medium were sub-cultured by streaking onto Xylose Lysine Deoxycholate (XLD) agar and incubated at 37 °C for 18–24 h. Colonies suspected to be *Salmonella* (1–3 colonies) on XLD agar, appearing red with black centers, were further identified by using biochemical tests on Triple Sugar Iron (TSI) agar and Lysine Indole Motility (LIM) medium. The biochemical profile for *Salmonella* was as follows: TSI, alkaline (red), slant and acid (yellow), gas producing (+), hydrogen sulfide (H_2_S) production (+); LIM, lysine-positive, motility-positive, and indole-negative, in accordance with WHO guidelines [21].

Serogroups of identified *Salmonella* isolates were determined by slide agglutination according to the Kauffmann–White scheme [22] using the commercial polyvalent O antisera (S&A Reagents Lab, Bangkok, Thailand). To identify the *Salmonella* serogroups, the *Salmonella* culture was suspended in one drop of polyvalent O-antisera OMA, OMB, OMC, OMD, OME, OMF, and OMG antisera in an orderly manner until a positive-agglutinated reaction was observed. Then, the identified polyvalent group isolate was further examined with the corresponding monovalent O group antisera in the positive polyvalent O group. All identified *Salmonella* serogroups were kept at −80 °C in glycerol broth for further analysis.

### 2.3. Antimicrobial Susceptibility Determination

A total of 338 *Salmonella* isolates were randomly selected for the antimicrobial susceptibility test. We selected two to three isolates from each canal per year, ensuring representation across the serogroup and the month of sampling. Antimicrobial susceptibility was determined against 13 agents belonging to 8 antimicrobial classes using the agar disk diffusion method, in accordance with guidelines by the Clinical and Laboratory Standards Institute (CLSI) [23]. The antimicrobials tested were as follows: ampicillin (AMP), 10 g; ceftazidime (CAZ), 30 μg; chloramphenicol (CHL), 30 μg; ciprofloxacin (CIP), 5 μg; ceftriaxone (CRO), 30 μg; cefotaxime (CTX), 30 μg; gentamicin (GEN), 10 μg; imipenem (IMP), 10 μg; nalidixic acid (NAL), 30 μg; norfloxacin (NOR), 10 μg; streptomycin (STR), 10 μg; sulfamethoxazole-trimethoprim (SXT), 23.75/1.25 μg; and tetracycline (TET), 30 μg. Pseudomonas aeruginosa ATCC 27853 and Escherichia coli ATCC 25922 were used as quality control strains. The inhibition zone diameter was measured, and the results were interpreted according to the CLSI guidelines. In the study area, commonly used antibiotics include ampicillin, third-generation cephalosporins, fluoroquinolones, aminoglycosides, tetracyclines, and sulfonamides. The 13 antimicrobials tested in this study cover most clinically relevant and commonly prescribed agents in Bangkok. Multi-drug resistance (MDR) was defined as resistance to one or more agents in at least three different antimicrobial classes [24].

### 2.4. Detection of ESBL Genes and Class 1 Integrons

*Salmonella* isolates that screened positive for ESBL, according to CLSI criteria, were examined for the presence of ESBL genes using multiplex polymerase chain reaction (PCR). Isolates were selected to minimize clonal duplication by excluding those with an identical serogroup, sampling site, and month of isolation. Three primer sets specific to *blaCTX-M*, *blaTEM,* and *blaSHV* were used, as previously designed [25]. It is acknowledged that certain *blaCTX-M, blaTEM,* and *blaSHV* variants represent non-ESBL β-lactamases; therefore, detection in this study reflects gene presence rather than functional confirmation of ESBL activity, as noted in the discussion. An initial annealing temperature gradient was performed to optimize the PCR conditions, after which all multiplex PCR assays were carried out in a final reaction volume of 25 μL containing 1 μL, alongside the DNA template, 12.5 μL of 2× Green Master Mix (New England Biolabs, MA 01938, USA), and 10 pmol of each gene-specific primer. The optimized cycling conditions were as follows: initial denaturation at 95 °C for 15 min; 30 cycles of denaturation at 94 °C for 30 s, annealing at 60 °C for 30 s, elongation at 72 °C for 2 min; followed by final extension at 72 °C for 10 min [25].

The presence of class 1 integrons in MDR *Salmonella* isolates was determined by PCR targeting the *intI1* gene. Each reaction mixture (25 μL) contained 1 μL of the DNA template, 12.5 μL of 2× Green Master Mix (New England Biolabs, MA 01938, USA), and 10 pmol of each primer. The cycling parameters were initial denaturation at 95 °C for 5 min; 30 cycles of denaturation at 94 °C for 60 s, annealing at 60 °C for 45 s, extension at 72 °C for 50 s; followed by a final extension at 72 °C for 5 min [26]. The PCR amplicons were separated by horizontal electrophoresis on a 1.2% agarose gel prepared in Tris-borate-EDTA (TBE) buffer and run at 120 V for 40 min. DNA bands were visualized under UV light and photographed using a UVITEC Fire Reader (Uvitec Ltd., Cambridge, CB4 0WS, UK).

## 3. Results

### 3.1. Salmonella Confirmation and Identification

**Figure 2:**
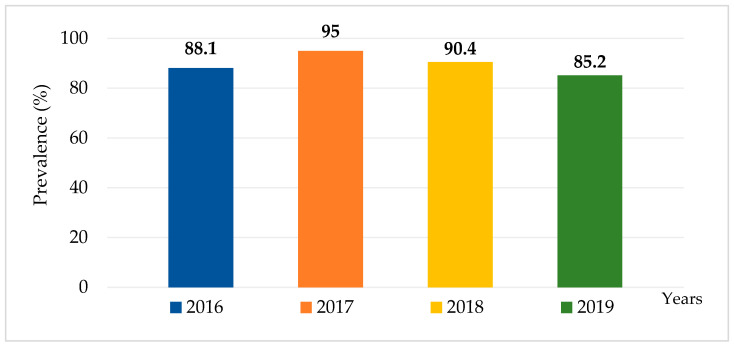
Prevalence of *Salmonella* contamination in canal water in Bangkok, Thailand, 2016–2019.

Over the four-year period from 2016 to 2019, a total of 1381 canal water samples were collected from 29 canals in the Bangkok Metropolitan. Out of these samples, 1239 (89.7%) were culture positive for *Salmonella*. The highest percentage of *Salmonella* contamination was observed in 2017, with 95% (321/338) of samples, followed by 2018 with 90.4% (416/460), 2016 with 88.1% (288/327), and 2019 with 85.2% (218/256) indicating a persistently high environmental burden of *Salmonella* over the study period (Figure 2).

From the 1239 positive samples, a total of 1748 *Salmonella* isolates were recovered from the 1239 culture-positive water samples for serogrouping. Twelve serogroups were identified, with serogroup B being the most prevalent (41%, namely 717/1748), followed by serogroup C (29.5%, 516/1748) and serogroup E (16.2%, 283/1748). The distribution of serogroups varied annually; detailed trends are presented in Supplementary Material Table S1.

### 3.2. Antimicrobial Resistance Profiles

Antimicrobial susceptibility testing was performed on 338 *Salmonella* isolates, selected through stratified random sampling (2–3 isolates per canal site per year) between 2016 and 2019 to represent geographic and temporal variation. Among these isolates, more than half (62.1%, 210/338) exhibited resistance to at least one antimicrobial, resulting in 65 resistant profiles; more details are reported in Supplementary Material Table S2. High resistance rates were observed for streptomycin (41.4%, 140/338), tetracycline (36.4%, 123/338), and ampicillin (35.8%, 121/338). Resistance to sulfamethoxazole–trimethoprim (24%, 81/338), ciprofloxacin (23.7%, 80/338), nalidixic acid (23.7%, 80/338), and chloramphenicol (14.2%, 48/338) was also noted. Notably, all isolates remained susceptible to imipenem. Of particular concern, over 23% of isolates were resistant to quinolones and fluoroquinolones, and 4.7% were resistant to 3rd generation cephalosporin, indicating the presence of ESBL strains in the aquatic environment (Table 1).

Overall, the *Salmonella* isolates exhibited various AMR profiles (altogether there are 63 patterns, see details in Annex 2), of which 54.8% (115/210) of the isolates were resistant to at least three classes of antibiotics, hence meeting the criteria for being MDR *Salmonella*. The prevalence of MDR *Salmonella* was highest in 2016 (71.2%, 37/52), followed by 2017 (51.7%, 31/60), 2018 (53.1%, 26/49), and 2019 (42.9%, 21/49), as seen in Supplementary Material Table S1. Furthermore, between 2016 and 2019, 51 isolates (24.3%, 51/210) were resistant to five or more antibiotic classes tested, indicating extensively drug-resistant (XDR) *Salmonella*, as shown in Table 2.

### 3.3. Prevalence of ESBL Genes and Class 1 Integrons in Salmonella Isolates

*Salmonella* isolates that demonstrate positive screening as ESBL-producing strains by CLSI criteria were investigated further by using Multiplex PCR to detect the presence of ESBL genes. The PCR products of *blaCTX-M*, *blaTEM,* and *blaSHV* genes were applied. In total, 18 ESBL *Salmonella* isolates were investigated for the presence of ESBL genes. We found that the suspected ESBL *Salmonella* carried *blaCTX-M* (72.2%, 13/18), *blaTEM* (66.7%, 12/18), and *blaSHV* (5.6%, 1/18). In addition, the ESBL-producing isolates also exhibited the presence of multiple types of ESBL genes, of which 10/18 (55.6%) were *blaCTX-M* + *blaTEM*. Moreover, all 115 isolates of integron were screened for class 1 integrons by the specific class 1 integrase gene (*intI1*) using PCR. The integrase gene was detected in 67.8% (78/115) of these *Salmonella* isolates. Of these, *Salmonella* isolated in 2016 (62.2%; 23/37), 2017 (67.7%; 21/31), 2018 (69.2%; 18/26), and 2019 (76.2%; 16/21) were positive for the *intI1* gene, with no significant difference across four years (Chi-squared test, *p* value = 0.74).

## 4. Discussion

### 4.1. AMR Challenges in Environment in Urban Setting

Bangkok, a densely populated city with over 10 million residents and 3503 inhabitants per square kilometer [27], has a massive network of canals that historically served as major routes for trade and transportation. However, rapid urbanization has led to environmental challenges, particularly water contamination due to inadequate wastewater management. Many residential and industrial areas release untreated sewage directly into canals, making these waterways reservoirs for AMR bacteria.

*Salmonella* is a major foodborne pathogen and a key contributor to AMR worldwide. Salmonellosis is globally recognized as one of the leading causes of acute human bacterial gastroenteritis [28]. It colonizes the gastrointestinal tract of humans and animals and is a primary indicator of fecal contamination in water, food, and the environment [29]. Moreover, as it has the ability to acquire and transfer resistance genes, particularly through horizontal gene transfer, *Salmonella* is considered a key marker for understanding the spread of AMR. Studying *Salmonella* provides insight into both its role in public health as a pathogen and its contribution to the overall AMR problem in environments.

Our findings indicate an alarmingly high prevalence of *Salmonella* in Bangkok’s urban canals, with the pathogen detected in 89.7% of water samples. This result indicates that these urban canals are significant reservoirs for bacterial pathogens. Similar findings have been reported in other rapidly urbanizing regions, such as Dhaka, Bangladesh, where high levels of bacterial contamination in urban rivers have been linked to untreated sewage disposal [30]. The widespread presence of *Salmonella* poses a serious public health risk, particularly in communities relying on canal water for agriculture, household activities, and recreation. The spread of *Salmonella* through urban environments highlights the urgent need for improving wastewater management to reduce bacterial contamination and address AMR.

This study also revealed high levels of AMR, with 62% of *Salmonella* isolates exhibiting resistance to at least one antibiotic. Resistance to streptomycin was most common (41.4%), consistent with the Suwannee River watershed in the Southeastern United States, where nearly all *Salmonella* isolates (98.9%) were resistant to streptomycin [31]. A systematic review and meta-analysis from Ethiopia reported the pooled proportion of streptomycin resistance of 47% (95% CI: 35–60%) [32]. Streptomycin is specifically used for the treatment of tuberculosis and is not commonly employed in other clinical practices [33]. Private pharmacies commonly dispense broad-spectrum antibiotics, including aminopenicillins and fluoroquinolones [34]. Streptomycin residues can exert selective pressure on bacterial populations, promoting the survival of resistant strains, which may transmit to humans via the food-chain system [32,35].

Of particular concern is the high prevalence of ESBL genes, particularly *blaCTX-M*, found in 72.2% of resistant isolates. This rate was slightly lower than 75% reported in northern Thailand [36], but it highlights the widespread occurrence of resistance mechanisms that undermine the effectiveness of beta-lactam antibiotics for treatment of serious infections. This observation is consistent with global reports identifying *blaCTX-M* as one of the most predominant ESBL genes in both clinical and environmental settings, reflecting its successful dissemination via mobile genetic elements. The presence of genes indicates that the bacteria have developed mechanisms to resist antibiotics that are commonly used to treat infections, thereby increasing the risk of treatment failure. The detection of *blaCTX-M* in the environment is a major concern as it can be horizontally transferred to other bacteria, a process that facilitates the emergence of multidrug resistance.

A study in Jordan reported a high prevalence of *Salmonella* resistant to tetracycline and multidrug-resistant *Salmonella* Typhimurium [37]. A systematic review and meta-analysis showed increasing prevalence of *Salmonella* to nalidixic acid and tetracycline in South Asia [38]. Further, Non-Typhoidal *Salmonella* (NTS) is a major cause of acute diarrhea with characteristic multidrug resistance (MDR), commonly reported in Asia [39].

### 4.2. MDR Challenges

Additionally, 54.8% of *Salmonella* isolates were MDR, which is lower than 80% reported from India [40] but higher than the 31.2% observed in the southeastern United States [41]. These variations in MDR prevalence may be attributed to differences in antibiotic regulations, wastewater treatment, and antibiotic use policies across countries. Antibiotic usage in agriculture is high in India, and the prevalence of MDR bacteria is also higher. In contrast, stricter regulations in some Western countries might contribute to lower rates of MDR *Salmonella*.

Furthermore, 67.8% of the MDR *Salmonella* isolates contained class 1 integrons, which are genetic elements that further facilitate the capture, integration, and expression of ARGs. Class 1 integrons are particularly significant because they are frequently associated with plasmids and transposons, enabling their mobility across diverse bacterial hosts and environments. They play a key critical role in the spread and persistence of AMR in the environment by promoting the acquisition and dissemination of resistance genes. The co-occurrence of *blaCTX-M* and class 1 integrons in a substantial proportion of isolates indicates a heightened potential for co-selection and maintenance of multiple resistance determinants in canal water ecosystems. These findings suggest that urban canals in Bangkok act as important environmental reservoirs for AMR genes, promoting their rapid spread among microbial communities. Similar studies in China have emphasized the role of integrons in the persistence of AMR in aquatic environments [42], underlining the urgent need for targeted interventions in environmental settings.

### 4.3. Weak Regulation in Environmental Sector

This highlights how policy and regulation play significant roles in determining the spread of AMR in the environment. The high prevalence of MDR *Salmonella* in Bangkok’s canals is closely linked to poor wastewater management. Many densely populated communities lack proper facilities for effective management of sewage, especially in congested communities in Bangkok. In 2023, out of 2.69 million cubic meters (0.95 million tonnes per year) of household sewage, only 16.7% were properly treated by well-designed 238 treatment facilities. Only 40% of municipalities have legally mandated facilities to manage sewage, and the management of sewage by sub-district local governments remains unclear [43]. Similar challenges have been reported in Nairobi, Kenya, where inadequate waste disposal practices have led to microbial contamination of water sources [44]. The continuous contamination of human and animal waste into the water systems contributes significantly to the persistence of bacteria, including resistant bacteria in the urban environment.

Furthermore, the presence of antibiotic residues in wastewater is a selective pressure for the emergence of AMR bacteria. Studies from India have shown that untreated wastewater containing antibiotic residues creates selective pressure, allowing resistant bacteria to thrive [36]. This situation allows AMR bacteria, including *Salmonella*, to survive and proliferate, leading to an increased risk of foodborne infections and treatment failures.

Addressing AMR in the environment requires urgent policy interventions. Despite Thailand’s National Strategic Plan on AMR (2017–2022), which has concluded, and the current second National Action Plan on AMR (2023–2027), environmental AMR surveillance remains underdeveloped [7]. AMR control efforts have largely focused on human and animal health, neglecting the environmental dimension. The result of this study highlights the urgent need for new policies to address AMR contamination in Bangkok’s canal water.

International examples such as Sweden have successfully lowered AMR levels by implementing strict antibiotic stewardship programs, thereby improving wastewater treatment infrastructure and facilitating effective regulation [45,46]. Implementing similar measures in Thailand could significantly reduce the spread of resistant bacteria. Multi-sectoral collaboration between public health and agriculture authorities, environmental agencies, and academic institutions is key to sustainable AMR mitigation in the environment.

### 4.4. Limitations of This Study

This study did not conduct phenotypic testing for ESBL production or PCR-based detection of *Salmonella* spp., which may limit the interpretation of functional resistance expression and species-level confirmation. Nevertheless, the findings provide valuable insights into the prevalence of antimicrobial resistance genes in canal water over four years. Future studies that include both phenotypic confirmation and molecular detection are recommended to strengthen the conclusions and advance an understanding of resistance dynamics in aquatic environments.

Despite the correlation between antibiotic residues and the abundance of antibiotic-resistant genes in environmental samples [47], this study did not quantify antibiotic residues in canal water, which could provide additional insights into the selective pressures driving AMR. Future work should integrate chemical analysis of residues with microbiological surveillance to better understand AMR emergence and persistence in aquatic environments.

This study was also limited by its geographic scope, its focus on a single bacterial species (*Salmonella*), the lack of integration of relevant environmental parameters, and the absence of intervention assessment. Future studies should address these limitations by expanding the geographic coverage, including multiple bacterial species, incorporating environmental factors, and evaluating the impact of interventions to provide a more comprehensive understanding of AMR in urban water environments. In addition, this study focused exclusively on *Salmonella* in canal water in Bangkok, which limits the generalizability of the findings to other regions, to other bacterial species, or to other water sources that may also contribute to AMR. To capture a broader picture, future research could apply advanced techniques, such as metagenomics, to detect a wider range of AMR genes across diverse environmental settings. Furthermore, assessing the influence of additional environmental drivers—such as heavy metal pollution—on the emergence and spread of AMR would enhance our understanding of the underlying dynamics. A nationwide study that assesses AMR in various water systems across Thailand would be invaluable for guiding policymakers in developing more effective AMR control measures. Finally, establishing long-term monitoring of AMR trends in urban waterways would also provide insights into the effectiveness of wastewater treatment improvements in reducing AMR bacteria.

## 5. Conclusions

The high prevalence of AMR *Salmonella* in Bangkok’s urban canals underscores the urgent need for improved wastewater management and stronger environmental AMR monitoring. Without effective interventions in managing effluent from households, hospitals, and animal farms before releasing this to the environment, these waterways will continue to serve as reservoirs and transmission pathways for AMR bacteria, threatening public health.

We recommend three synergistic policies. First, strengthening regular AMR monitoring in water sources will help to identify contamination hotspots. Second, implementing effective and affordable treatment of household sewage and enforced by municipality regulations, effluent from public and private hospitals, and animal farms regulated by the Ministry of Public Health and the Ministry of Agriculture and Cooperatives. Third, regular monitoring of the quality of effluents from the human and animal sectors for improvement of sewage management and other relevant policy actions.

## Figures and Tables

**Table 1:** Number and percentage of *Salmonella* isolates from canal water in Bangkok, Thailand, which are resistant to antibiotics.

Year *(samples)*	Number (%) of AMR *Salmonella* Isolates												
	AMP	CAZ	CRO	CTX	IMP	GEN	STR	TET	CIP	NAL	NOR	SXT	CHL
2016 *(n = 84)*	35 (41.7)	4 (4.8)	6 (7.1)	6 (7.1)	0 (0)	6 (7.1)	35 (41.7)	36 (42.9)	30 (35.7)	23 (27.4)	2 (2.4)	21 (25)	18 (21.4)
2017 *(n = 87)*	30 (34.5)	0 (0)	2 (2.3)	2 (2.3)	0 (0.)	6 (6.9)	36 (41.4)	34 (39.1)	26 (29.9)	21 (24.1)	3 (3.4)	20 (23)	15 (17.2)
2018 *(n = 86)*	26 (30.2)	4 (4.7)	4 (4.7)	5 (5.8)	0 (0)	5 (5.8)	36 (41.9)	27 (31.4)	16 (18.6)	18 (20.9)	1 (1.2)	19 (22.1)	11 (12.8)
2019 *(n = 81)*	30 (37)	2 (2.5)	3 (3.7)	3 (3.7)	0 (0)	5 (6.2)	33 (40.7)	26 (32.1)	8 (9.8)	18 (22.2)	0 (0)	21 (25.9)	4 (4.9)
Total *(n = 338)*	121 (35.8)	10 (3)	15 (4.4)	16 (4.7)	0 (0)	22 (6.5)	140 (41.4)	123 (36.4)	80 (23.7)	80 (23.7)	6 (1.8)	81 (24)	48 (14.2)

Abbreviations: ampicillin (AMP), ceftazidime (CAZ), ceftriaxone (CRO), cefotaxime (CTX), imipenem (IMP), gentamicin (GEN), chloramphenicol (CHL), ciprofloxacin (CIP), streptomycin (STR), tetracycline (TET), nalidixic acid (NAL), norfloxacin (NOR), sulfamethoxazole-trimethoprim (SXT).

**Table 2:** Number and percentages of *Salmonella* isolates resistant to 3–7 classes of antibiotics, 2016–2019.

Year *(Isolates)*	Number (%) of MDR *Salmonella* Isolates					Total MDR
	3 Classes	4 Classes	5 Classes	6 Classes	7 Classes	
2016 *(n = 52)*	11 (21.1)	9 (17.3)	11 (21.1)	5 (9.6)	1 (1.9)	37 (71.2)
2017 *(n = 60)*	9 (15)	11 (18.3)	8 (13.3)	3 (5)	0 (0)	31 (51.7)
2018 *(n = 49)*	6 (12.2)	6 (12.2)	9 (18.4)	3 (6.1)	2 (4.1)	26 (53.1)
2019 *(n = 49)*	3 (6.1)	9 (18.4)	5 (10.2)	2 (4.1)	2 (4.1)	21 (42.9)
Total *(n = 210)*	29 (13.8)	35 (16.7)	33 (15.7)	13 (6.2)	5 (2.4)	115 (54.8)

## Data Availability

The data presented in this study are available on request from the corresponding author due to privacy reasons.

## Supplementary Materials

The following supporting information can be downloaded at https://www.mdpi.com/article/10.3390/ijerph22091333/s1, Table S1: Prevalence of AMR *Salmonella* spp.; Table S2: AMR profiles.

## Abbreviations

The following abbreviations are used in this manuscript:

AMP	Ampicillin
AMR	Antimicrobial resistance
ARGs	Antibiotic resistance genes
CAZ	Ceftazidime
CFU/L	Colony forming unit per liter
CHL	Chloramphenicol
CIP	Ciprofloxacin
CLSI	Clinical and Laboratory Standards Institute
CRO	Ceftriaxone
CTX	Cefotaxime
°C	Degree Celsius
DNA	Deoxyribonucleic acid
ESBL	Extended-spectrum β-lactamase
FQs	Fluoroquinolones
GEN	Gentamicin
H2S	Hydrogen sulfide
*intI1*	Class 1 integrase gene
IMP	Imipenem
LIM	Lysine Indole Motility
μg	microgram
μL	microliter
MDR	Multidrug resistance
MSRV	Modified semisolid Rappaport Vasiliadis
NAL	Nalidixic acid
NOR	Norfloxacin
pmol	Picomole
PCR	Polymerase chain reaction
STR	Streptomycin
SXT	Sulfamethoxazole-trimethoprim
TET	Tetracycline
TSI	Triple Sugar Iron
WHO	World Health Organization
XDR	Extensively drug-resistant
XLD	Xylose Lysine Deoxycholate

## References

[1] Salam M.A., Al-Amin M.Y., Salam M.T., Pawar J.S., Akhter N., Rabaan A.A., Alqumber M.A.A. (2023). Antimicrobial Resistance: A Growing Serious Threat for Global Public Health. Healthcare.

[2] World Health Organization (2020). GLASS Whole-Genome Sequencing for Surveillance of Antimicrobial Resistance. https://www.who.int/publications/i/item/9789240011007.

[3] Aslam B., Wang W., Arshad M.I., Khurshid M., Muzammil S., Rasool M.H., Nisar M.A., Alvi R.F., Aslam M.A., Qamar M.U. (2018). Antibiotic resistance: A rundown of a global crisis. Infect. Drug Resist..

[4] Mackenzie J.S., Jeggo M. (2019). The One Health Approach-Why Is It So Important?. Trop. Med. Infect. Dis..

[5] World Health Organization (2020). World Leaders Join Forces to Fight the Accelerating Crisis of Antimicrobial Resistance. https://www.who.int/news/item/20-11-2020-world-leaders-join-forces-to-fight-the-accelerating-crisis-of-antimicrobial-resistance.

[6] Nadimpalli M., Astagneau E.D., Love D.C., Price L.B., Huynh B.-T., Collard J.-M., Lay K.S., Borand L., Ndir A., Walsh T.R. (2018). Bacterial Infections and antibiotic-Resistant Diseases among Young Children in low-income countries (BIRDY) Study Group. Combating Global Antibiotic Resistance: Emerging One Health Concerns in Lower- and Middle-Income Countries. Clin. Infect. Dis..

[7] Thailand National Strategic Plan on Antimicrobial Resistance 2017–2021. https://amrthailand.net/.

[8] Koutsoumanis K., Allende A., Álvarez-Ordóñez A., Bolton D., Bover-Cid S., Chemaly M., Davies R., De Cesare A., Herman L., EFSA Panel on Biological Hazards (BIOHAZ) (2021). Role played by the environment in the emergence and spread of antimicrobial resistance (AMR) through the food chain. EFSA J..

[9] Michael I., Rizzo L., McArdell C.S., Manaia C.M., Merlin C., Schwartz T., Dagot C., Fatta-Kassinos D. (2013). Urban wastewater treatment plants as hotspots for the release of antibiotics in the environment: A review. Water Res..

[10] Mezal E.H., Sabol A., Khan M.A., Ali N., Stefanova R., Khan A.A. (2014). Isolation and molecular characterization of *Salmonella* enterica serovar Enteritidis from poultry house and clinical samples during 2010. Food Microbiol..

[11] Lianou A., Panagou E.Z., Nychas G.J.E. (2017). Chapter 17—Meat Safety—I Foodborne Pathogens and Other Biological Issues. Lawrie’s Meat Science.

[12] Chahal C., van den Akker B., Young F., Franco C., Blackbeard J., Monis P. (2016). Pathogen and particle associations in wastewater significance and implications for treatment and disinfection processes. Adv. Appl. Microbiol..

[13] Liu H., Whitehouse C.A., Li B. (2018). Presence and Persistence of *Salmonella* in Water: The Impact on Microbial Quality of Water and Food Safety. Front. Public Health.

[14] Collier S.A., Deng L., Adam E.A., Benedict K.M., Beshearse E.M., Blackstock A.J., Bruce B.B., Derado G., Edens C., Fullerton K.E. (2021). Estimate of Burden and Direct Healthcare Cost of Infectious Waterborne Disease in the United States. Emerg. Infect. Dis..

[15] World Health Organization (2022). Drug-Resistant Salmonella. http://www.who.int/mediacentre/factsheets/fs139/en/.

[16] Lekagul A., Kirivan S., Tansakul N.N., Krisanaphan C., Srinha J., Laoprasert T., Kaewkhankhaeng W., Tangcharoensathien V., Odetokun I.A. (2023). Antimicrobial consumption in food-producing animals in Thailand between 2017 and 2019: The analysis of national importation and production data. PLoS ONE.

[17] Lekagul A., Tangcharoensathien V., Mills A., Rushton J., Yeung S. (2020). How antibiotics are used in pig farming: A mixed- methods study of pig farmers, feed mills and veterinarians in Thailand. BMJ Glob. Health.

[18] Legakul A., Yeung S., Tangcharoensathien V. (2019). Patterns of antibiotic use in global pig production: A systematic review. Vet. Anim. Sci..

[19] Manyi-Loh C.M.S., Meyer E., Okoh A. (2018). Antibiotic Use in Agriculture and Its Consequential Resistance in Environmental Sources: Potential Public Health Implications. Molecules.

[20] World Health Organization (2017). Guidelines for Drinking-Water Quality.

[21] (2015). WHO GFN Laboratory Protocol: “Biochemical Identification of *Salmonella* and Shigella, Using an Abbreviated Panel of Tests”—Version 002. https://www.researchgate.net/publication/242575055_Biochemical_Identification_of_Salmonella_and_Shigella_Using_an_Abbreviated_Panel_of_Tests.

[22] WHO Collaborating Centre for Reference and Research on *Salmonella* (2007). Antigenic Formulae of the Salmonella Serovars.

[23] CLSI (2020). Performance Standards for Antimicrobial Susceptibility Testing.

[24] Magiorakos A.-P., Srinivasan A., Carey R.B., Carmeli Y., Falagas M.E., Giske C.G., Harbarth S., Hindler J.F., Kahlmeter G., Olsson-Liljequist B. (2012). Multidrug-resistant, extensively drug-resistant and pandrug-resistant bacteria: An international expert proposal for interim standard definitions for acquired resistance. Clin. Microbiol. Infect..

[25] Monstein H.J., Balkhed A.O., Nilsson M.V., Dornbusch K., Nilsson L.E. (2007). PCR amplification assay for the detection of *bla*SHV, *bla*TEM and *blaCTX-M* genes in Enterobacteriaceae. APMIS.

[26] Ebrahim-Saraie H.S., Nezhad N.Z., Heidari H., Motamedifar A., Motamedifar M. (2018). Detection of Antimicrobial Susceptibility and Integrons Among Extended-spectrum β-lactamase Producing Uropathogenic *Escherichia coli Isolates* in Southwestern Iran. Oman Med. J..

[27] Population Density in Bangkok, Thailand from 2014 to 2023. https://www.statista.com/statistics/1422857/thailand-population-density-in-bangkok/.

[28] Galán-Relaño Á., Díaz A.V., Lorenzo B.H., Gómez-Gascón L., Rodríguez M.Á.M., Jiménez E.C., Rodríguez F.P., Márquez R.J.A. (2023). *Salmonella* and Salmonellosis: An Update on Public Health Implications and Control Strategies. Animals.

[29] Mohammad M.B., Saydur Rahman M. (2024). *Salmonella* in the environment: A review on ecology, antimicrobial resistance, seafood contaminations, and human health implications. J. Hazard. Mater. Adv..

[30] Amin N., Rahman M., Raj S., Ali S., Green J., Das S., Doza S., Mondol M.H., Wang Y., Islam M.A. (2019). Quantitative assessment of fecal contamination in multiple environmental sample types in urban communities in Dhaka, Bangladesh using SaniPath microbial approach. PLoS ONE.

[31] Luo Z., Gu G., Ginn A., Giurcanu M.C., Adams P., Vellidis G., van Bruggen A.H.C., Danyluk M.D., Wright A.C., Griffiths M.W. (2015). Distribution and Characterization of *Salmonella enterica* Isolates from Irrigation Ponds in the Southeastern United States. Appl. Environ. Microbiol..

[32] Mengistu G., Dejenu G., Tesema C., Arega B., Awoke T., Alemu K., Moges F., Clegg S. (2020). Epidemiology of streptomycin resistant *Salmonella* from humans and animals in Ethiopia: A systematic review and meta-analysis. PLoS ONE.

[33] Clinical Practice Guideline (CPG) of Tuberculosis Treatment in Thailand. https://www2.si.mahidol.ac.th/km/download/20796/?tmstv=1739241009.

[34] Siltrakool B., Berrou I., Griffiths D. (2021). Alghamdi. Antibiotics’ Use in Thailand: Community Pharmacists’ Knowledge, Attitudes and Practices. Antibiotics.

[35] Miller S.A., Ferreira J.P., LeJeune J.T. (2022). Antimicrobial Use and Resistance in Plant Agriculture: A One Health Perspective. Agriculture.

[36] Assawatheptawee K., Tansawai U., Kiddee A., Thongngen P., Punyadi P., Romgaew T., Kongthai P., Sumpradit T., Niumsup P.R. (2017). Occurrence of Extended-Spectrum and AmpC-Type β-Lactamase Genes in *Escherichia coli* Isolated from Water Environments in Northern Thailand. Microbes Environ..

[37] Burjaq S.Z., Abu-Romman S.M. (2020). Prevalence and Antimicrobial Resistance of *Salmonella* spp. From Irrigation Water in Two Major Sources in Jordan. Curr. Microbiol..

[38] Talukder H., Roky S.A., Debnath K., Sharma B., Ahmed J., Roy S. (2023). Prevalence and Antimicrobial Resistance Profile of *Salmonella* Isolated from Human, Animal and Environment Samples in South Asia: A 10-Year Meta-analysis. J. Epidemiol. Glob. Health.

[39] Sharma N.C., Kumar D., Sarkar A., Chowdhury G., Mukhopadhyay A.K., Ramamurthy T. (2020). Prevalence of Multidrug Resistant Salmonellae with Increasing Frequency of *Salmonella enterica* Serovars Kentucky and Virchow among Hospitalized Diarrheal Cases in and around Delhi, India. Jpn. J. Infect. Dis..

[40] Kapley A., Sheeraz M.S., Kukade S., Ansari A., Qureshi A., Bajaj A., Khan N.A., Tandon S., Jain R., Dudhwadkar S. (2023). Antibiotic resistance in wastewater: Indian scenario. Environ. Pollut..

[41] Velasquez C.G., Macklin K.S., Kumar S., Bailey M., Ebner P., Oliver H., Martin-Gonzalez F., Singh M. (2021). Prevalence and antimicrobial resistance of *Salmonella* isolated from poultry in the southeastern United States. Poult. Sci..

[42] Han N., Sheng D., Xu H. (2012). Role of *Escherichia coli* strain subgroups, integrons, and integron-associated gene cassettes in dissemination of antimicrobial resistance in aquatic environments of Jinan, China. Water Sci. Technol..

[43] Project to Develop and Promote Toilet and Waste Management for Safety in Sanitation and Health (In Thai). https://env.anamai.moph.go.th/th/kpi68/download?id=123434&mid=39402&mkey=m_document&lang=th&did=35932.

[44] Mohamed S.A., Nyerere, Sang W.K., Ngayo M. (2021). Bottled water brands are contaminated with multidrug resistant bacteria in Nairobi, Kenya. F1000Research.

[45] Mölstad S., Löfmark S., Carlin K., Erntell M., Aspevall O., Blad L., Hanberger H., Hedin K., Hellman J., Norman C. (2017). Lessons learnt during 20 years of the Swedish strategic programme against antibiotic resistance. Bull. World Health Organ..

[46] Swedish Strategy to Combat Antibiotic Resistance 2024–2025. https://www.government.se/contentassets/1fedc516373d421f919814f1963e2fe1/amr_strategi_eng_web_ny2.pdf.

[47] Larsson D.G.J., Flach C.F. (2022). Antibiotic resistance in the environment. Nat. Rev. Microbiol..

