# Supplementary material for: Antimicrobial Resistant Salmonella in Canal Water in Bangkok, Thailand: Survey Results Between 2016 and 2019
Source: Int J Environ Res Public Health. 2025 Aug 27;22(9):1333. doi: 10.3390/ijerph22091333 (PMC12469579; doi:10.3390/ijerph22091333)
Supplement: Supplementary file 1 [file ijerph-22-01333-s001.zip › Table 2. AMR profiles.pdf]

Table 2. of AMR profile of *Salmonella* isolates from canal water in Bangkok, Thailand between 2016 and 2019

No	Resistance patterns	No. of resistance isolates				Total (%)
		**2016**	**2017**	**2018**	**2019**	
	**Single class**	**11**	**20**	**18**	**17**	**66 (31.4)**
1	AMP		1	2		3 (4.5)
2	CIP	1	2	2		5 (7.6)
3	GEN				1	1 (1.5)
4	NAL		1	1	3	5 (7.6)
5	STR	5	11	12	10	38 (57.6)
6	SXT				1	1 (1.5)
7	TET	1	2			3 (4.5)
8	CIP-NAL	4	2	1	2	9 (13.6)
9	CIP-NAL-NOR		1			1 (1.5)
	**Double classes**	**4**	**9**	**5**	**11**	**29 (13.8)**
10	AMP-NAL			1	1	2 (6.9)
11	AMP-TET	1	2	1	5	9 (31.0)
12	AMP-STR				1	1 (3.4)
13	AMP-SXT				2	2 (6.9)
14	CIP-STR		1			1 (3.4)
15	NAL-STR			1		1 (3.4)
16	AMP-GEN-STR				1	1 (3.4)
17	AMP-CIP-NAL	1	1			2 (6.9)

No	Resistance patterns	No. of resistance isolates				Total (%)
		2016	2017	2018	2019	
18	CHL-CIP-NAL	1	3			4 (13.8)
19	CIP-NAL-SXT			1	1	2 (6.9)
20	CIP-NAL-TET	1	2			3 (10.3)
21	CIP-NAL-NOR-STR			1		1 (3.4)
	**Triple classes**	**11**	**9**	**6**	**3**	**29 (13.8)**
22	AMP-CHL-TET			1		1 (3.4)
23	AMP-STR-TET	4	1	2	3	10 (34.5)
24	AMP-SXT-TET	2	2			4 (13.8)
25	AMP-CRO-CTX-CHL	2				2 (6.9)
26	AMP-NAL-NOR-CIP-TET		1			1 (3.4)
27	STR-CIP-TET		1			1 (3.4)
28	STR-NAL-CIP/TET	1	3	1		5 (17.2)
29	SXT-NAL-TET			1		1 (3.4)
30	SXT-NAL-CIP-TET	2		1		3 (10.3)
31	SXT-STR-GEN-CHL		1			1 (3.4)
	**Quadruple classes**	**9**	**11**	**6**	**8**	**35 (16.7)**
32	AMP-GEN-CHL-TET	1				1 (2.9)
33	AMP-STR-CHL-TET	1				1 (2.9)
34	AMP-STR-CIP-TET			1		1 (2.9)
35	AMP-SXT-CHL-TET	1	1			2 (5.7)

No	Resistance patterns	No. of resistance isolates				Total (%)
		2016	2017	2018	2019	
36	AMP-SXT-NAL-TET		1			1 (2.9)
37	AMP-SXT-STR-TET	2	6	4	6	18 (51.4)
38	AMP-STR-GEN-CHL-TET	1				1 (2.9)
39	AMP-SXT-NAL-CIP-TET				1	1 (2.9)
40	AMP-STR-GEN-CHL-NAL-CIP	2	2			4 (11.4)
41	AMP-STR-GEN-NAL-NOR-CIP-TET		1			1 (2.9)
42	CTX-SXT-NAL-CIP-TET			1		1 (2.9)
43	STR-CHL-NAL-CIP-TET	1				1 (2.9)
44	SXT-STR-NAL-TET				1	1 (2.9)
45	SXT-STR-NAL-CIP-TET				1	1 (2.9)
	**Quintuple classes**	**11**	**8**	**9**	**5**	**33 (15.7)**
46	AMP-SXT-CHL-CIP-TET		1	1		2 (6.1)
47	AMP-SXT-STR-CHL-CIP	1				1 (3)
48	AMP-SXT-STR-CHL-TET	1	3	3		7 (21.2)
49	AMP-SXT-STR-NAL-TET		1	2	3	6 (18.2)
50	AMP-SXT-STR-NAL-CIP-TET	9	2	2	2	15 (45.5)
51	AMP-CRO-CTX-STR-GEN-CHL-TET		1	1		2 (6.1)
	**Sextuple classes**	**5**	**3**	**3**	**2**	**13 (6.2)**
52	AMP-SXT-STR-CHL-CIP-TET	1	2	1		4 (30.8)
53	AMP-CRO-CTX-GEN-CHL-CIP-TET		1			1 (7.7)

No	Resistance patterns	No. of resistance isolates				Total (%)
		2016	2017	2018	2019	
54	AMP-SXT-STR-GEN-CHL-NAL-TET			1		1 (7.7)
55	AMP-SXT-STR-CHL-NAL-CIP-TET				1	1 (7.7)
56	AMP-SXT-STR-CHL-NOR-CIP-TET	1				1 (7.7)
57	AMP-CAZ-CRO-CTX-STR-CHL-CIP-TET	2				2 (15.4)
58	AMP-CRO-CTX-SXT-STR-GEN-CHL-TET				1	1 (7.7)
59	AMP-CAZ-CRO-CTX-STR-GEN-CHL-NAL-CIP-TET			1		1 (7.7)
60	AMP-CAZ-CRO-CTX-STR-GEN-CHL-NAL-NOR-CIP-TET	1				1 (7.7)
	**Septuple classes**	**1**	**0**	**2**	**2**	**5 (2.4)**
61	AMP-CRO-CTX-SXT-STR-GEN-CHL-NAL-TET				1	1 (20)
62	AMP-CRO-CTX-SXT-STR-GEN-CHL-NAL-CIP-TET				1	1 (20)
63	AMP-CAZ-CRO-CTX-SXT-STR-GEN-CHL-NAL-CIP-TET	1		2		3 (60)
	**Total**	**52**	**60**	**49**	**49**	**210 (100)**
